# Eight new species of *Otacilia* (Araneae: Phrurolithidae) from southern China

**DOI:** 10.3897/zookeys.979.56273

**Published:** 2020-10-27

**Authors:** Ke-ke Liu, Yuan-hao Ying, Yu-xin Xiao, Jing Yan, Yong-hong Xiao

**Affiliations:** 1 College of Life Science, Jinggangshan University, Ji’an 343009, Jiangxi, China Jinggangshan University Ji'an China

**Keywords:** Jiangxi Province, sac spider, Taxonomy

## Abstract

Eight new *Otacilia* species were collected from Ji’an City, Jiangxi Province, China during a survey of the phrurolithid fauna of the region: *Otacilia bizhouica* Liu, **sp. nov.** (♂♀), *O. gougunao* Liu, **sp. nov.** (♂), *O. nanhuashanica* Liu, **sp. nov.** (♂♀), *O. subfabiformis* Liu, **sp. nov.** (♂♀), *O. wugongshanica* Liu, **sp. nov.** (♂♀), *Otacilia yusishanica* Liu, **sp. nov.** (♂♀), *O. zaoshiica* Liu, **sp. nov.** (♂♀) and *O. ziyaoshanica* Liu, **sp. nov.** (♀). All species are described and illustrated with photographs and SEM micrographs, and their distribution is also mapped.

## Introduction

During the past five years, the total number of phrurolithid species recorded from China has almost doubled, with most of the newly discovered species being endemic to the country (WSC 2015–2019). All of them (47 species) were discovered in southern China and most belong to the genus *Otacilia* Thorell, 1897 (WSC 2020). However, there are still many poorly known *Otacilia* species from southern China with unusual morphological characteristics.

*Otacilia* is the most diverse of the 15 phrurolithid genera (WSC 2020). In recent major reviews of the genus, 27 species *Phrurolithus* C.L. Koch, 1839 were transferred to *Otacilia* (Zamani & Marusik, 2020) and one new genus, *Aboculus* Liu, 2020, was erected (Liu et al. 2020). Recently, seven new species and one new combination were recorded from Jinggang Mountain National Nature Reserve in Jiangxi Province, which is the first report on phrurolithid spiders from this province (Liu et al. 2020). Their distribution also implies that *Otacilia* species may be abundant in this province.

When we focused on sac spiders in the Jiangxi Province of southern China, many unknown *Otacilia* species with unusual characters were found. Therefore, eight new *Otacilia* species were identified and are described here.

## Materials and methods

Specimens were examined using a Zeiss Stereo Discovery V12 stereomicroscope with a Zoom Microscope System. Both male palps and female copulatory organs were dissected and examined in 75% ethanol, using a Zeiss Axio Scope A1 compound microscope with a KUY NICE CCD. The epigynes were cleared with pancreatin solution. Specimens, including dissected male palps and epigynes, were stored in 80% ethanol after examination. All the specimens are deposited in Animal Specimen Museum, College of Life Science, Jinggangshan University (**ASM-JGSU**).

The measurements were taken with ImageView CM2000 software and are given in millimetres. The body length of all specimens excludes the chelicerae and spinnerets. Terminology of the male and female genitalia follows Jäger and Wunderlich (2012), Ramírez (2014), Jäger and Dimitrov (2019), Liu et al. (2019) and Zamani and Marusik (2020). Promarginal and retromarginal teeth on the chelicerae are given as the first, second, third, etc., and measured from the base of the fang to the distal groove.

Leg measurements are given as total length (femur, patella, tibia, metatarsus, tarsus). Leg spines are documented by dividing each leg segment into two aspects: prolateral (p) and retrolateral (r) and indicating the ventral (v) spines as single (1) or paired (2), e.g., femur I pv1111; tibia I v2222. Dorsal spines on femora are recorded separately.

The abbreviations used in the text are as follows:

**Eyes**

**ALE** = anterior lateral eye

**AME** = anterior median eye

**MOA** = median ocular area

**PLE** = posterior lateral eye

**PME** = posterior median eye

**Male palp**

**DTA** = dorsal tibial apophysis

**dTA** = distal tegular apophysis

**E** = embolus

**FA** = femoral apophysis

**Gr** = groove

**RTA** = retrolateral tibial apophysis

**rTA** = retrolateral tegular apophysis

**SD** = sperm duct

**VTA** = ventral tibial apophysis

**Epigyne**

**B** = bursa

**CD** = copulatory duct

**CO** = copulatory opening

**CT** = connecting tube

**FD** = fertilization duct

**GA** = glandular appendage

**MS** = median septum

**Spe** = spermathecae

## Taxonomy

### Family Phrurolithidae Banks, 1892

**Genus *Otacilia* Thorell, 1897**

#### Otacilia bizhouica

Taxon classificationAnimaliaAraneaePhrurolithidae

Liu sp. nov.

602D69BB-BF06-5FB9-970A-C5B4DE502F03

http://zoobank.org/3B75F002-AA06-4D64-A017-56DEA6E52213

**Figure 1.**
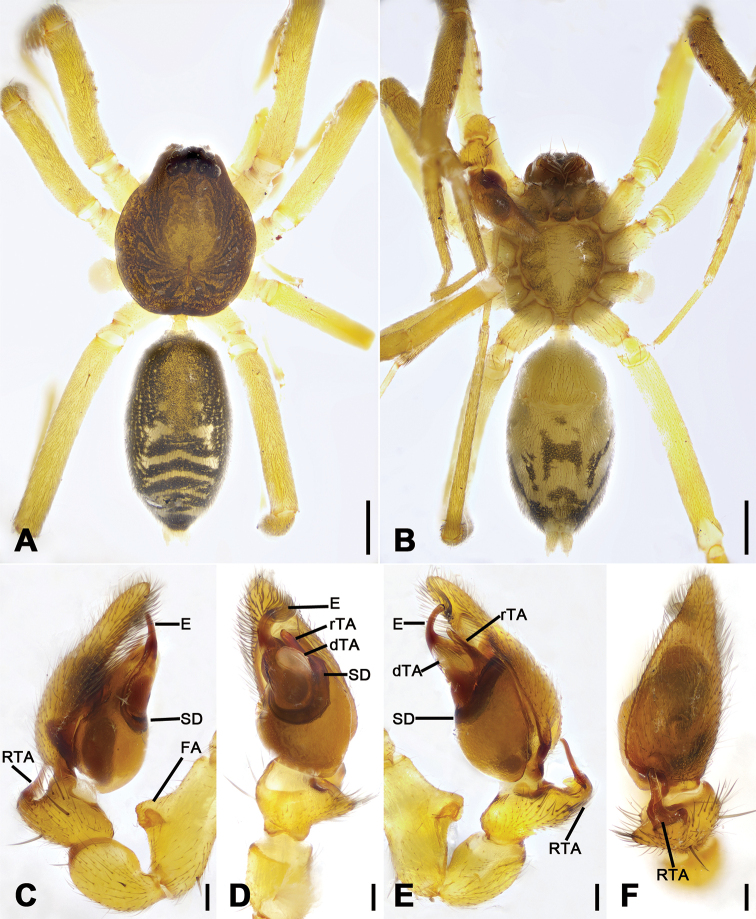
*Otacilia bizhouica* sp. nov., male holotype. **A** habitus, dorsal view **B** same, ventral view **C** palp, prolateral view **D** same, ventral view **E** same, retrolateral view **F** same, dorsal view. Scale bars: 0.5 mm (**A, B**), 0.1 mm (**C–F**). Abbreviations: dTA – distal tegular apophysis, E – embolus, FA – femoral apophysis, RTA – retrolateral tibial apophysis, rTA – retrolateral tegular apophysis, SD – sperm duct.

**Figure 2.**
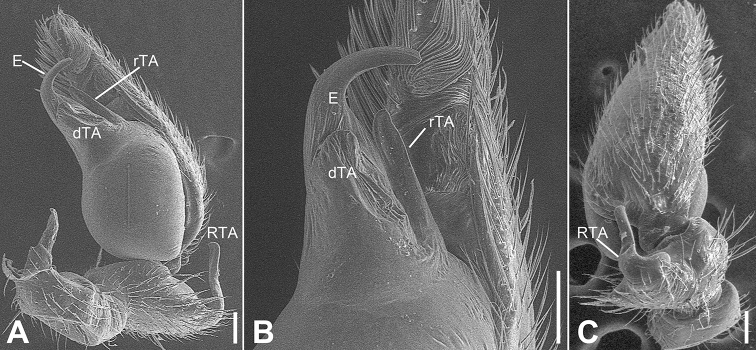
SEM micrographs of *Otacilia bizhouica* sp. nov., palp of male holotype. **A** ventro-retrolateral view **B** same, detail of embolus, distal tegular apophysis and retrolateral tegular apophysis **C** dorsal view, detail of retrolateral tibia apophysis. Scale bars: 0.1 mm. Abbreviations: dTA – distal tegular apophysis, E – embolus, RTA – retrolateral tibial apophysis, rTA – retrolateral tegular apophysis.

**Figure 3.**
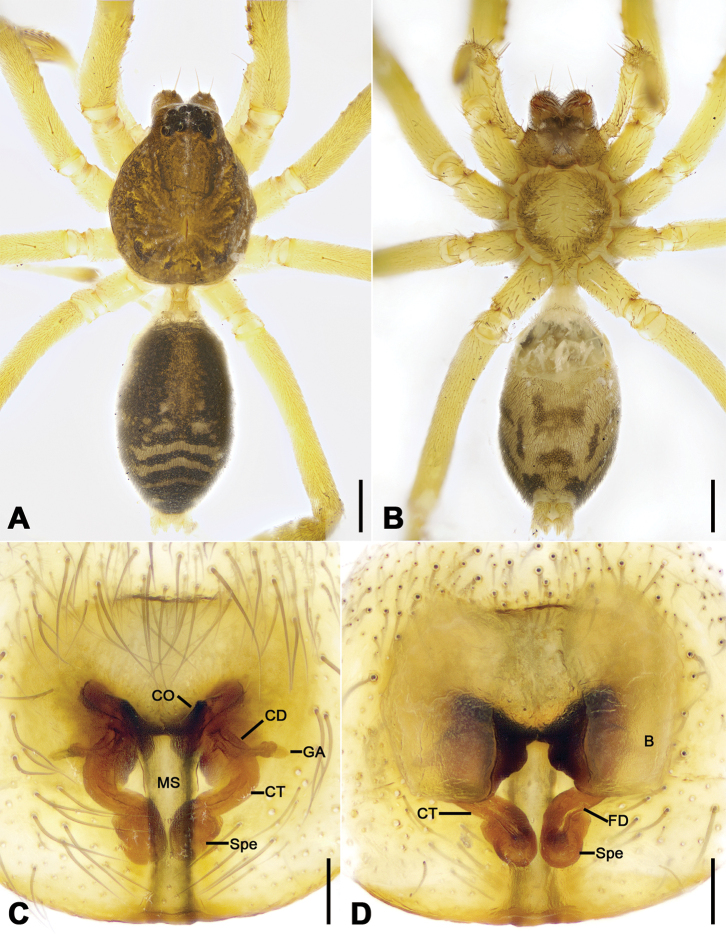
*Otacilia bizhouica* sp. nov., female paratype. **A** habitus, dorsal view **B** same, ventral view **C** epigyne, ventral view **D** epigyne, dorsal view. Scale bars: 0.5 mm (**A, B**), 0.1 mm (**C, D**). Abbreviations: B – bursa, CD – copulatory duct, CO – copulatory opening, CT – connecting tube, FD – fertilization ducts, GA – glandular appendage, MS – median septum, Spe – spermathecae.

Figures 1 , 2 , 3 , 22

##### Type material.

***Holotype***: ♂, China, Jiangxi Province, Ji’an City, Suichuan county, Bizhou Town, Baishuixian Village, Dakeng Group, 26°19'55.98"N, 114°44'08.72"E, 362 m, 4 October 2019, leg. Ke-ke Liu et al. ***Paratypes***: 2 ♀, with the same data as holotype.

##### Etymology.

The specific name derived from the type locality, Bizhou Town; adjective.

##### Diagnosis.

The male of the new species is similar to *Otacilia liupan* Hu & Zhang, 2011 in having a short retrolateral tibial apophysis bending inwards to the base of the cymbium and the sub-circular sperm duct (see Hu and Zhang 2011: 60, figs 2−5), but can be separated from it by the thick retrolateral tegular apophysis (Figs 1D, E, 2A, B) (vs. thin) and a stubby pipe-shaped retrolateral tibial apophysis in dorsal view (Figs 1E, F, 2C) (vs. finger-like). The females resemble those of *O. ovoidea* Liu, 2020 in having sclerotized epigynal ridges (Fig. 3C, D), but can be separated from it by the rectangular median septum (vs. funnel-shaped) and the U-shaped spermathecae (vs. globular) (see Liu et al. 2020: 22, fig. 14C, D).

##### Description.

Male (Holotype). Habitus as in Fig. 1A, B. Total length 3.56, carapace 1.56 long, 1.33 wide. Eye sizes and interdistances: AME 0.09, ALE 0.10, PME 0.08, PLE 0.09; ALE−AME 0.01, AME-AME 0.05, PLE−PME 0.06, PME-PME 0.12, ALE−ALE 0.24, PLE−PLE 0.38, ALE−PLE 0.07, AME−PME 0.10, AME−PLE 0.16. MOA 0.25 long, frontal width 0.21, posterior width 0.26. Chelicerae (Fig. 1A, B) with three promarginal (proximal largest, distal smallest) and six retromarginal teeth (distal largest, 5^th^ smallest). Sternum (Fig. 1B), posterior end triangular, relatively blunt. Pedicel 0.2 long. Abdomen (Fig. 1A, B) 1.80 long, 1.06 wide. Leg measurements: I 6.46 (1.75, 0.58, 2.03, 1.53, 0.57); II 5.28 (1.32, 0.56, 1.55, 1.21, 0.64); III 4.39 (1.08, 0.42, 1.09, 1.16, 0.64); IV 7.31 (1.94, 0.61, 1.75, 2.01, 1.00). Leg spination (Fig. 1A, B): femur I with two dorsal spines; femora II–IV with one dorsal spine each; femora I pv1111, II pv111; tibiae I v22222222, II v2222222; metatarsi I v2222, II v222.

Colouration (Fig. 1A, B). Carapace yellow-brown, with radial, irregular dark yellow-brown mottled markings on surface. Chelicerae yellow-brown. Endites yellow, mottled. Sternum yellow, lateral margins with dark mottled markings. Legs yellow. Abdomen yellow-brown, with pair of large triangular yellowish spots on posterior dorsal scutum, three light chevron-shaped stripes on sub-medial part, and yellowish arc-shaped stripe posteriorly; weak dorsal scutum in anterior half; venter with H-shaped and pair of sloping markings posteriorly.

Palp (Figs 1C−F, 2). Femoral apophysis well-developed, as wide as half of femoral length. Patella unmodified. Tibia with large retrolateral apophysis, as long as tibial length, apex blunt, bending inwards to base of cymbium, with a broad base and a basal apophysis, directed dorsally in dorsal view. Cymbium width less than half of its length. Bulb broad oval, with sub-circular sperm duct, apophyses absent. Embolus hook-shaped, thick, with broad triangular base. Retrolateral tegular apophysis straight, thick, submedial part covered by distal tegular apophysis. Distal tegular apophysis oval, arising from base of embolus and retrolateral sperm duct.

Female (paratype). Habitus as in Fig. 3A, B. Lighter than male. Total length 4.04, carapace 1.68 long, 1.45 wide. Eye sizes and interdistances: AME 0.08, ALE 0.09, PME 0.09, PLE 0.08, AME−AME 0.05, AME−ALE 0.02, PME−PME 0.13, PME−PLE 0.06, AME−PME 0.05, AME−PLE 0.17, ALE−ALE 0.23, PLE−PLE 0.37, ALE−PLE 0.1. MOA 0.25 long, frontal width 0.20, posterior width 0.27. Chelicerae (Fig. 3A, B) with three promarginal (proximal largest, distal smallest) and five retromarginal teeth (distal largest, fourth smallest). Pedicel 0.3 long. Abdomen (Fig. 3A) 2.05 long, 1.2 wide. Leg (Fig. 3A, B) measurements: I 6.35 (1.53, 0.48, 2.25, 1.41, 0.68); II 5.83 (1.52, 0.63, 1.68, 1.30, 0.70); III 4.66 (1.21, 0.51, 1.06, 1.18, 0.70); IV 7.32 (1.97, 0.57, 1.72, 2.11, 0.95). Leg spination (Fig. 3A, B): tibiae I v222222222, II v2222222.

Epigyne (Fig. 3C, D). Epigynal plate funnel-shaped, posterior with elongate rectangular median septum. Copulatory ducts, glandular appendages, connecting tubes and spermathecae distinctly visible through integument in intact epigyne. Anterior fovea separated by weakly sclerotized transverse margin, medially with V-shaped sclerotized plug, covering copulatory openings. Copulatory ducts broad, short, posteriorly with pair of kidney-shaped transparent bursae medially. Glandular appendages relatively long, located on anterior of copulatory ducts, extending postero-laterally. Connecting tubes short, broad, as long as copulatory ducts, located between glandular appendages and spermathecae, posteriorly close to each other. Spermathecae U-shaped, anterior part slightly separated, posterior part touching. Fertilization duct short, located sub-medially on spermathecae, directed anterolaterally.

##### Distribution.

Known only from the type locality in Jiangxi Province, China (Fig. 22).

#### Otacilia gougunao

Taxon classificationAnimaliaAraneaePhrurolithidae

Liu sp. nov.

B22630D0-9B30-5FC4-8F5B-03F892C4ED7B

http://zoobank.org/20022BC6-E020-4F2E-B8D0-8CF2C88B81A1

Figures 4 , 5 , 22

##### Type material.

***Holotype***: ♂, China, Jiangxi Province, Ji’an City, Suichuan county, Nanjiang Town, Xiajiaoling Village, 26°00'39.41"N, 114°01'03.91"E, 979 m, 5 October 2019, leg. Ke-ke Liu et al. ***Paratypes***: 3 ♂, with same data as holotype.

##### Etymology.

The specific name refers to a famous tea from the type locality, Gougunao, which is planted on the mountainsides of Suichuan County; noun in apposition.

##### Diagnosis.

**Figure 4.**
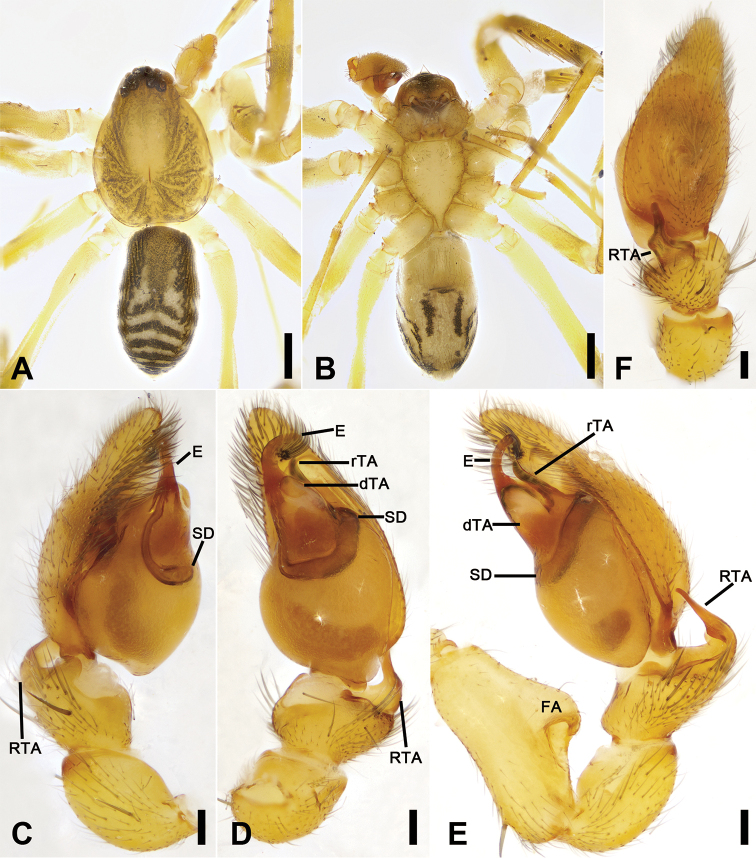
*Otacilia gougunao* sp. nov., male holotype. **A** habitus, dorsal view **B** same, ventral view **C** palp, prolateral view **D** same, ventral view **E** same, retrolateral view **F** same, dorsal view, slightly retrolateral. Scale bars: 0.5 mm (**A, B**), 0.1 mm (**C–F**). Abbreviations: dTA – distal tegular apophysis, E – embolus, rTA – retrolateral tegular apophysis, RTA – retrolateral tibial apophysis, SD – sperm duct.

**Figure 5.**
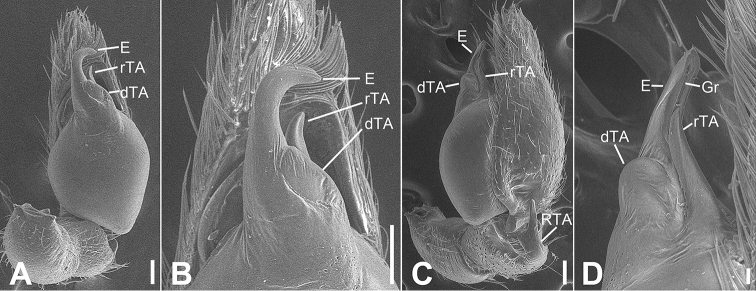
SEM micrographs of *Otacilia gougunao* sp. nov., palp of male holotype. **A** ventral view **B** same, detail of embolus, distal tegular apophysis and retrolateral tegular apophysis **C** same, retro-dorsolateral view **D** same, detail of embolus, embolic groove, distal tegular apophysis and retrolateral tegular apophysis. Scale bars: 0.1 mm (**A–C**), 20 µm (**D**). Abbreviations: dTA – distal tegular apophysis, E – embolus, Gr – groove, RTA – retrolateral tibial apophysis, rTA – retrolateral tegular apophysis.

The males of the new species resemble those of *O. bizhouica* sp. nov. in having an ovoid membranous distal tegular apophysis and hook-shaped embolus (Fig. 1C−F), but can be distinguished from it by the sternum with a sharpened end (Fig. 4B) (vs. relatively blunt), the retrolateral tibial apophysis with a submedial apophysis prolaterally (Figs 4E, F, 5C) (vs. with a basal apophysis prolaterally) and the retrolateral tegular apophysis with a slightly curved apex (Figs 4D, E, 5) (vs. straight apex).

##### Description.

Male (holotype). Habitus as in Fig. 4A, B. Total length 3.49, carapace 1.87 long, 1.44 wide. Eye sizes and interdistances: AME 0.09, ALE 0.09, PME 0.08, PLE 0.09, AME−AME 0.05, AME−ALE 0.03, PME−PME 0.14, PME−PLE 0.07, AME−PME 0.1, AME−PLE 0.18, ALE−ALE 0.26, PLE−PLE 0.41, ALE−PLE 0.13. MOA 0.25 long, frontal width 0.22, posterior width 0.29. Chelicerae (Fig. 4B) with three promarginal (proximal largest, distal smallest) and six retromarginal teeth (distal largest, proximal smallest, others equal in size). Sternum posteriorly pointed. Pedicel 0.10 long. Abdomen (Fig. 4A, B), 1.67 long, 1.00 wide. Leg measurements (Fig. 4A, B): I 6.46 (1.66, 0.57, 2.12, 1.42, 0.69); II 5.07 (1.21, 0.54, 1.59, 1.05, 0.68); III 4.81 (1.25, 0.50, 1.03, 1.29, 0.74); IV 7.74 (2.41, 0.62, 1.71, 2.21, 0.79). Leg spination (Fig. 4A, B): femur I with two dorsal spines, femora II−IV with one dorsal spine each; femora I pv1111, pv111 (right), II pv111; tibiae I v2222222, II v2222222; metatarsi I v2222, II v2222.

Colouration (Fig. 4A, B). Carapace yellow, with radial, irregular dark stripes submarginally and arc-shaped dark stripes around margin. Chelicerae yellow-brown. Endites and labium yellow, mottled. Legs yellow. Abdomen dark brown, with pair of racket-shaped yellowish spots at posterior of dorsal scutum, three light chevron-shaped stripes on sub-medial part, and two yellowish arc-shaped stripes posteriorly; weak dorsal scutum in anterior half; venter with N-shaped marking and pair sloping markings posteriorly.

Palp (Figs 4C−F, 5). Femoral apophysis well-developed, width more than half of femoral length. Patella unmodified. Tibia with large retrolateral apophysis, less than tibial length, apex blunt, bending inwards to base of cymbium, with submedian apophysis prolaterally and basal apophysis retrolaterally. Cymbium width less than half of its length. Bulb broad oval, with U-shaped sperm duct, apophyses absent. Embolus hook-like, thick, with broad triangular base and narrowed groove, apart from retrolateral tegular apophysis and distal tegular apophysis. Retrolateral tegular apophysis clavate, thick, with slightly curved apex, directed anterolaterally, more than basal 2/3 covered by distal tegular apophysis in ventral view. Distal tegular apophysis oval, arising from base of embolus and retrolateral sperm duct.

Female. Unknown.

##### Distribution.

Known only from the type locality in Jiangxi Province, China (Fig. 22).

#### Otacilia nanhuashanica

Taxon classificationAnimaliaAraneaePhrurolithidae

Liu sp. nov.

954D7335-959A-5CA7-A2A0-CE2F248BD226

http://zoobank.org/34A24780-C0E2-4C27-BA43-5F89EF751B67

**Figure 6.**
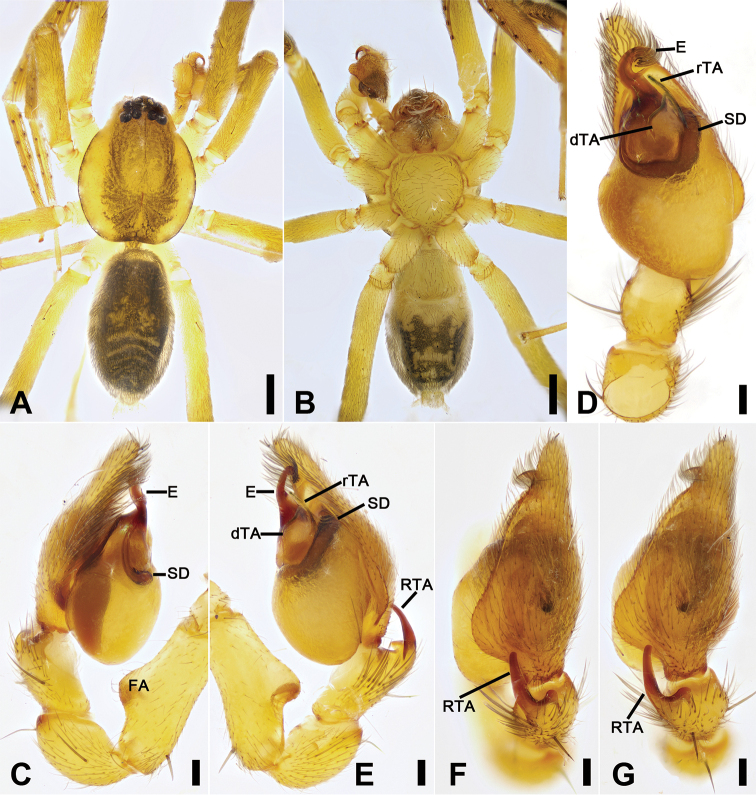
*Otacilia nanhuashanica* sp. nov., male holotype. **A** habitus, dorsal view **B** same, ventral view **C** palp, prolateral view **D** same, ventral view **E** same, retrolateral view **F** same, retro-dorsal view **G** same, dorsal view. Scale bars: 0.5 mm (**A, B**), 0.1 mm (**C–G**). Abbreviations: dTA – distal tegular apophysis, E – embolus, FA – femoral a pophysis, rTA – retrolateral tegular apophysis, RTA – retrolateral tibial apophysis, SD – sperm duct.

**Figure 7.**
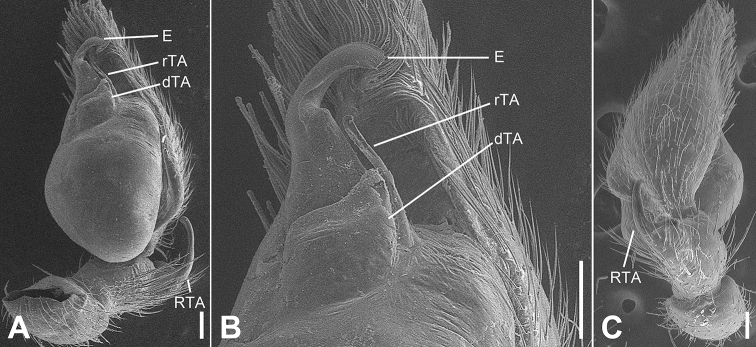
SEM micrographs of *Otacilia nanhuashanica* sp. nov., palp of male holotype. **A** ventro-retrolateral view **B** same, detail of embolus, distal tegular apophysis and retrolateral tegular apophysis **C** same, dorsal view. Scale bars: 0.1 mm (**A–C**). Abbreviations: dTA – distal tegular apophysis, E – embolus, RTA – retrolateral tibial apophysis, rTA – retrolateral tegular apophysis.

**Figure 8.**
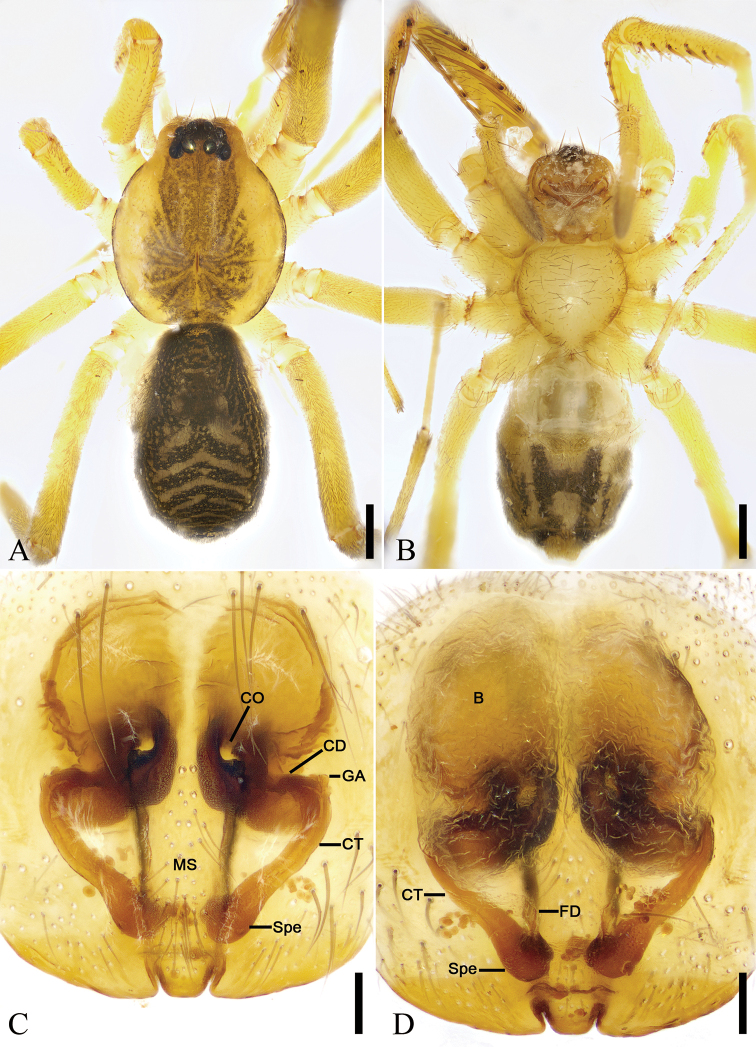
*Otacilia nanhuashanica* sp. nov., female paratype. **A** habitus, dorsal view **B** same, ventral view **C** epigyne, ventral view **D** epigyne, dorsal view. Scale bars: 0.5 mm (**A, B**), 0.1 mm (**C, D**). Abbreviations: B – bursa, CD – copulatory duct, CO – copulatory opening, CT – connecting tube, FD – fertilization ducts, GA – glandular appendage, MS – median septum, Spe – spermathecae.

Figures 6 , 7 , 8 , 22

##### Type material.

***Holotype***: ♂, China, Jiangxi Province, Ji’an City, Yongxin County, Nanhua Mt., 26°50'22.02"N, 114°15'47.05"E, 1130 m, 3 October 2019, leg. Ke-ke Liu et al. ***Paratypes***: 1 ♂, 2 ♀, with same data as holotype; 1 ♂ (right palp broken in collection), Zhongcun, 26°49'37.77"N, 114°13'14.55"E, 3 October 2019, leg. Ke-ke Liu et al.

##### Etymology.

The specific name is derived from the type locality, Nanhuashan; adjective.

##### Diagnosis.

The males of the new species are similar to *Otacilia hengshan* (Song, 1990) in having a hook-shaped embolus, semi-circular sperm duct and a clavate retrolateral tegular apophysis (see Hu and Zhang 2011: 62, fig. 9−11), but can be separated from it by the embolus with a trapezoid base (Figs 6D, 7A, B) (vs. parallel-sided) and the thin clavate retrolateral tegular apophysis (Figs 6D, E, 7A, B) (vs. thick). The females resemble *O. hengshan* in having narrow and convergent connecting tubes (see Hu and Zhang 2011: 62, fig. 13, 15), but can be separated from it by the epigyne with a broad sub-trapezoid median septum (Fig. 8C) (vs. slender).

##### Description.

Male (holotype). Habitus as in Fig. 6A, B. Total length 3.63, carapace 1.79 long, 1.51 wide. Eye sizes and interdistances: AME 0.11, ALE 0.09, PME 0.08, PLE 0.1, AME−AME 0.06, AME−ALE 0.02, PME−PME 0.13, PME−PLE 0.08, AME−PME 0.12, AME−PLE 0.18, ALE−ALE 0.31, PLE−PLE 0.44, ALE−PLE 0.1. MOA 0.30 long, frontal width 0.27, posterior width 0.30. Chelicerae (Fig. 6A, B) with three promarginal (middle largest, distal smallest) and five retromarginal teeth (distal largest, fourth smallest, first to third equal in size). Sternum (Fig. 6B) with small triangular, blunt end. Abdomen (Fig. 6A, B) 1.90 long, 1.08 wide. Leg measurements (Fig. 6A, B): I 7.31 (1.76, 0.67, 2.21, 1.75, 0.92); II 6.03 (1.54, 0.59, 1.71, 1.35, 0.84); III 4.75 (1.07, 0.57, 1.19, 1.20, 0.72); IV 7.78 (2.14, 0.62, 1.85, 2.14, 1.03). Leg spination (Fig. 6A, B): femur I with two dorsal spines, femora II−IV with one dorsal spine each; femora I pv1111, II pv11, pv111 (right); tibiae I v22222222, II v2222222; metatarsi I v2222, II pv2222.

Colouration (Fig. 6A, B). Carapace yellow-brown, medially with radial, irregular dark brown mottled markings on surface and arc-shaped dark stripes around margin. Fovea distinct, black. Chelicerae yellow-brown. Endites and labium yellow, with abundant setae on surface. Legs yellow. Abdomen dark brown, with pair of large irregular spots on posterior of dorsal scutum, three light chevron-shaped stripes on sub-medial part, and yellowish arc-shaped stripe posteriorly; weak dorsal scutum in anterior half; venter with two pairs of W-shaped markings posteriorly.

Palp (Figs 6C−F, 7). Femoral apophysis well-developed, width longer than half of its length. Patella unmodified. Retrolateral tibial apophysis less than tibial length, bending inward to base of cymbium, with clear apophysis located retrolaterally at base and blunt apex in dorsal view. Sperm duct C-shaped, strongly sclerotized, around base of retrolateral tegular apophysis, distal tegular apophysis and embolus. Retrolateral tegular apophysis clavate, longer than distal tegular apophysis. Distal tegular apophysis ampulla-like, covering half of retrolateral tegular apophysis. Embolus with trapezoidal base and short hook-like tip.

Female (paratype). Habitus as in Fig. 8A, B. Total length 3.91, carapace 1.84 long, 1.61 wide. Eye sizes and interdistances: AME 0.1, ALE 0.11, PME 0.09, PLE 0.09, AME−AME 0.06, AME−ALE 0.02, PME−PME 0.12, PME−PLE 0.06, AME−PME 0.10, AME−PLE 0.19, ALE−ALE 0.29, PLE−PLE 0.42, ALE−PLE 0.11. MOA 0.26 long, front width 0.23, posterior width 0.31. Chelicerae (Fig. 8A, B) with three promarginal (middle largest, distal smallest) and six retromarginal teeth (distal largest, proximal smallest, second to fourth equal in size, 5^th^ and 6^th^ with a same base). Abdomen (Fig. 14A, B) 2.03 long, 1.25 wide. Legs (Fig. 8A, B) measurements: I 7.77 (1.94, 0.72, 2.45, 1.84, 0.82); II 6.36 (1.63, 0.63, 1.78, 1.64, 0.68); III 5.31 (1.44, 0.60, 1.15, 1.32, 0.80); IV 8.26 (2.29, 0.69, 1.98, 2.22, 1.08). Leg spination (Fig. 8A, B): femora I−IV with one dorsal spine each; femora I p11111, p1111(right), II p111; tibiae I v22222222, II v22222222.

Epigyne (Fig. 8C, D). Epigynal plate mask-shaped, sub-medially with pair of oval copulatory openings, posteriorly with sub-trapezoidal median septum. Copulatory ducts, glandular appendages, connecting tubes and spermathecae distinctly visible through integument in intact epigyne. Copulatory ducts relatively broad, located between copulatory openings and glandular appendages, posteriorly with pair of large bean-shaped transparent bursae. Glandular appendages very short, partly covered by bursae, located on anterior of connecting tubes. Connecting tubes longer than copulatory ducts, converging postero-medially, located between glandular appendages and spermathecae. Spermathecae slightly expanded, separated by less width of septum, directed medially. Fertilization duct short, directed anteriorly.

##### Distribution.

Known only from the type locality in Jiangxi Province, China (Fig. 22).

#### Otacilia subfabiformis

Taxon classificationAnimaliaAraneaePhrurolithidae

Liu sp. nov.

AA0C25D8-9DB9-59F0-91F3-9B9A05C0CF35

http://zoobank.org/1EA8C0FA-3A76-4C55-8149-BAB6E83B446F

**Figure 9.**
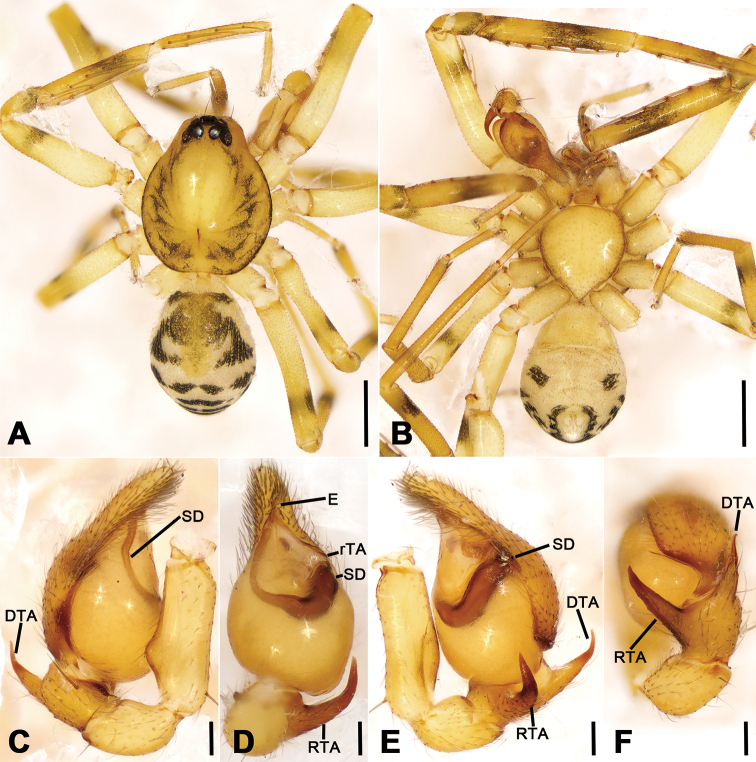
*Otacilia subfabiformis* sp. nov., male holotype. **A** habitus, dorsal view **B** same, ventral view **C** palp, prolateral view **D** same, ventral view **E** same, retrolateral view **F** same, dorsal view. Scale bars: 0.5 mm (**A, B**), 0.1 mm (**C–F**). Abbreviations: DTA– dorsal tibial apophysis, dTA – distal tegular apophysis, E – embolus, FA – femoral apophysis, RTA – retrolateral tibial apophysis, rTA – retrolateral tegular apophysis, SD – sperm duct.

**Figure 10.**
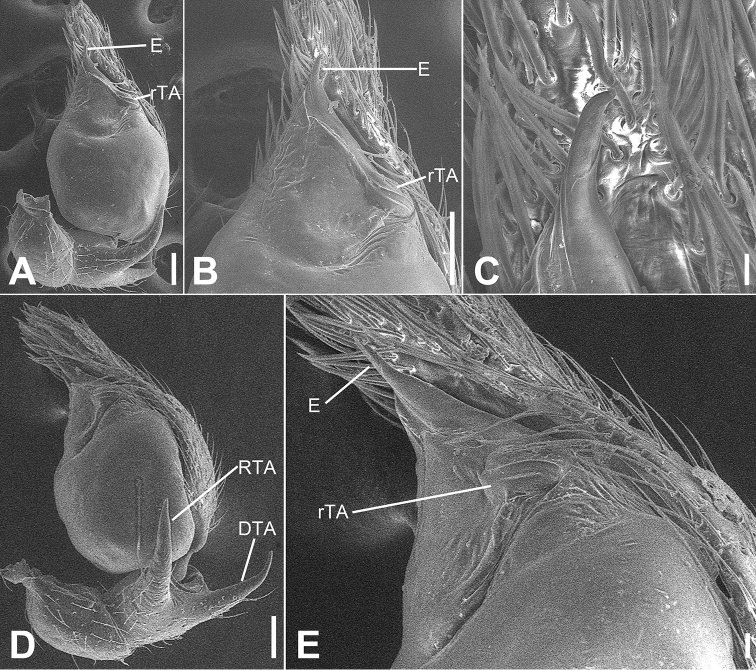
SEM micrographs of *Otacilia subfabiformis* sp. nov., palp of male holotype. **A** ventral view **B** same, detail of embolus and retrolateral tegular apophysis **C** same, detail of embolus **D** retrolateral view **E** same, detail of embolus and retrolateral tegular apophysis. Scale bars: 0.1 mm (**A, B, D**), 10 µm (**C**), 20 µm (**E**). Abbreviations: DTA – dorsal tibial apophysis, E – embolus, FA – femoral apophysis, RTA – retrolateral tibial apophysis, rTA – retrolateral tegular apophysis.

**Figure 11.**
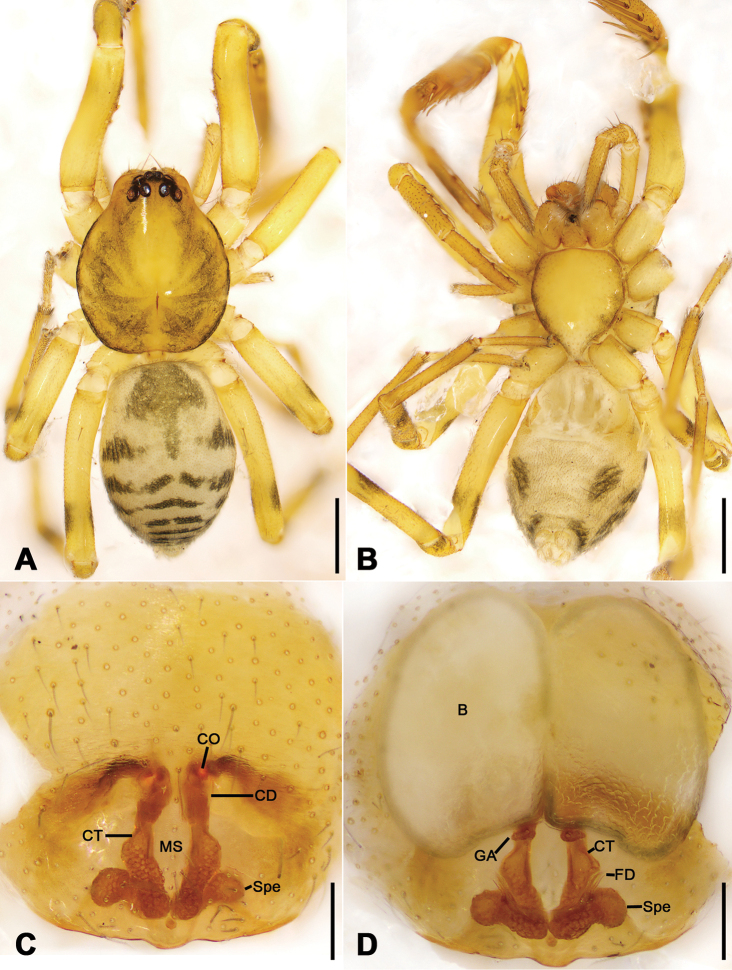
*Otacilia subfabiformis* sp. nov., female paratype. **A** habitus, dorsal view **B** same, ventral view **C** epigyne, ventral view **D** epigyne, dorsal view. Scale bars: 0.5 mm (**A, B**), 0.1 mm (**C, D**). Abbreviations: B – bursa, CD – copulatory duct, CO – copulatory opening, CT – connecting tube, FD – fertilization ducts, GA – glandular appendage, MS – median septum, Spe – spermathecae.

Figures 9 , 10 , 11 , 22

##### Type material.

***Holotype***: ♂, China, Jiangxi Province, Ji’an City, Anfu County, Taishan Town, Wugong Mt., near the ticket office, 27°27'10.79"N, 114°11'8.24"E, 4 January 2020, leg. Ke-ke Liu et al. ***Paratypes***: 1 ♂, 1 ♀, with same data as holotype.

##### Etymology.

The specific name is derived from that of a similar species, *O. fabiformis*Liu et al. 2019; adjective.

##### Diagnosis.

The males of the new species are similar to *Otacilia fabiformis* Liu, Xu, Xiao, Yin & Peng, 2019 in having a spine-like embolus, a C-shaped sperm duct and a swollen bulb (see Liu et al. 2019: 444, fig. 6C), but can be separated from it by the retrolateral tibial apophysis with a sharp apex (Figs 9E, F, 10D) (vs. with a blunt tip), the retrolateral tegular apophysis with a thin retrolateral part (Figs 9D, E, 10A, B, E) (vs. with broad retrolateral part). The female resembles *O. fabiformis* in having large and touching bursae (see Liu et al. 2019: 444, fig. 7C), but can be separated from it by the slightly curved and separated connecting tubes medially located (Fig. 11D) (vs. the strongly curved and separated connecting tubes laterally located).

##### Description.

Male (holotype). Habitus as in Fig. 9A, B. Total length 2.39, carapace 1.18 long, 1.01 wide. Eye sizes and interdistances: AME 0.05, ALE 0.08, PME 0.07, PLE 0.07, AME−AME 0.02, AME−ALE 0.02, PME−PME 0.06, PME−PLE 0.04, AME−PME 0.07, AME−PLE 0.12, ALE−ALE 0.14, PLE−PLE 0.29, ALE−PLE 0.05. MOA 0.19 long, frontal width 0.12, posterior width 0.21. Chelicerae (Fig. 9A, B) with three promarginal (proximal largest, distal smallest) and two retromarginal teeth (distal larger). Sternum (Fig. 9B) with small triangular, blunt end. Pedicel 0.13 long. Abdomen (Fig. 9A, B) 1.24 long, 0.84 wide. Leg measurements: I 4.36 (1.17, 0.40, 1.25, 1.09, 0.45); II 3.62 (0.91, 0.37, 0.96, 0.85, 0.36); III 3.45 (0.75, 0.26, 0.74, 0.83, 0.42); IV 3.79 (1.18, 0.36, 0.96, 1.29, 0.68). Leg spination (Fig. 9A, B): femora I−IV without dorsal spine each; femora I p111 II p11; tibiae I v222222, II v222222; metatarsi I v2222, II v222.

Colouration (Fig. 9A, B). Carapace yellow, with radial, irregular dark stripes mediolaterally and arc-shaped dark stripes around margin. Fovea distinct, black. Chelicerae, endites and labium yellow. Sternum yellow, margins with dark brown mottled spots. Legs yellow, femora I−IV each with black annulation; patellae I with black annulation; tibiae I with blackish-brown stripes, II−IV with blackish-brown annulations; metatarsi I−IV with blackish-brown annulations. Abdomen yellowish white, anteriorly with blackish-brown stripe, with round blackish-brown spots located in median dorsal scutum and pair of L-shaped blackish-brown stripes located at posterior of dorsal scutum, pair of oval blackish-brown spots on sub-median part, three blackish-brown stripes on posterior part; venter with pair of blackish-brown spots posterolaterally.

Palp (Figs 9C−F, 10). Femoral apophysis weakly sclerotized, width less than half of its length. Patella unmodified. Retrolateral tibial apophysis large, bending to posterior bulb, longer than tibia, with sharp apex. Dorsal tibial apophysis large, longer than tibia, with fine tip in retrolateral view. Sperm duct C-shaped, strongly sclerotized, around base of retrolateral tegular apophysis and embolus. Retrolateral tegular apophysis large, protruding retrolaterally, with narrow retrolateral part. Embolus short, spine-like.

Female (paratype). Habitus as in Fig. 11A, B. Total length 2.54, carapace 1.17 long, 1.02 wide. Eye sizes and interdistances: AME 0.06, ALE 0.07, PME 0.07, PLE 0.07, AME−AME 0.01, AME−ALE 0.01, PME−PME 0.06, PME−PLE 0.03, AME−PME 0.06, AME−PLE 0.11, ALE−ALE 0.28, PLE−PLE 0.37, ALE−PLE 0.05. MOA 0.18 long, frontal width 0.12, posterior width 0.20. Chelicerae (Fig. 11A, B) with three promarginal (proximal largest, distal smallest) and two retromarginal teeth (distal larger, with same base). Sternum (Fig. 11B) gradually pointed. Pedicel 0.10 long. Abdomen (Fig. 11A, B) 1.29 long, 0.92 wide. Leg measurements: I 4.07 (1.06, 0.42, 1.16, 1.02, 0.41); II 3.62 (0.80, 0.30, 1.06, 1.02, 0.44); III 2.98 (0.79, 0.34, 0.63, 0.75, 0.47); IV 4.21 (1.14, 0.36, 0.89, 1.21, 0.61). Leg spination (Fig. 11A, B): femur I with two dorsal spines, femora II, III, and IV with one dorsal spine each; femur I p111; tibiae I v2222222, II v222222; metatarsi I v2222, II v2222.

Colouration (Fig. 11A, B). Lighter than males. Abdomen, anteriorly with mushroom-like dark brown spot.

Epigyne (Fig. 11C, D). Epigynal plate tree-like, antero-medially with pair of concave copulatory openings, with sub-columnar median septum. Copulatory ducts, connecting tubes and spermathecae distinctly visible through integument in intact epigyne. Copulatory ducts relatively narrow, located between copulatory openings and glandular appendages, posteriorly with pair of large, bean-shaped, transparent bursae. Glandular appendages short, near base of bursae, located on anterior of connecting tubes. Connecting tubes slightly longer than copulatory ducts, located between glandular appendages and spermathecae, median part slightly expanded. Spermathecae globular peanut-shaped, touching. Fertilization duct long, anteriorly directed.

##### Distribution.

Known only from the type locality in Jiangxi Province, China (Fig. 22).

#### Otacilia wugongshanica

Taxon classificationAnimaliaAraneaePhrurolithidae

Liu sp. nov.

E7B979D5-7B8A-5FAE-958A-7A13CD7D5CD7

http://zoobank.org/5E87C18C-ADCF-4CB8-890F-2EF2EBD34B1C

**Figure 12.**
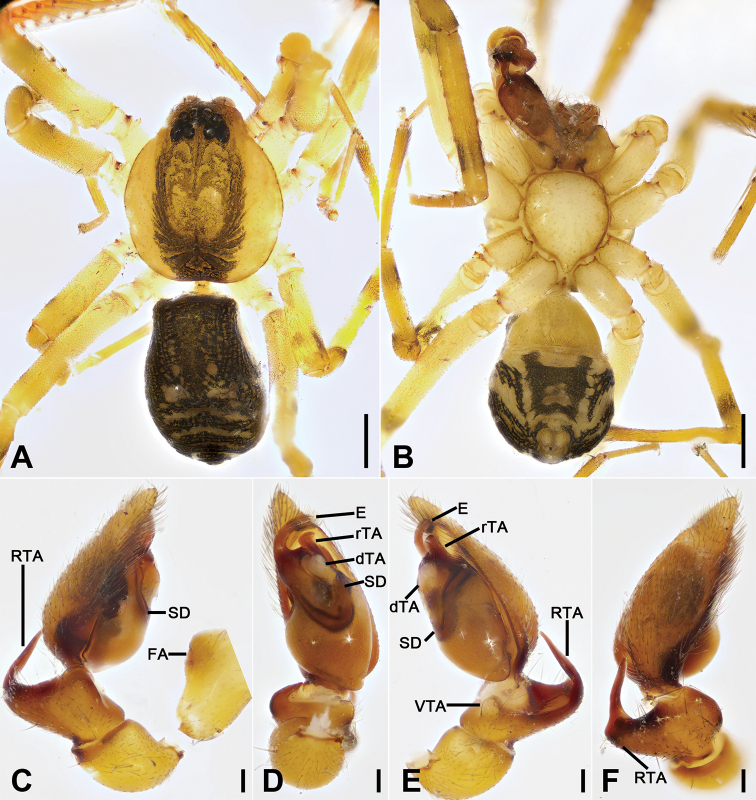
*Otacilia wugongshanica* sp. nov., male holotype. **A** habitus, dorsal view **B** same, ventral view **C** palp, prolateral view **D** same, ventral view **E** same, ventro-retrolateral view **F** same, dorsal view. Scale bars: 0.5 mm (**A, B**), 0.1 mm (**C–F**). Abbreviations: dTA – distal tegular apophysis, E – embolus, FA – femoral apophysis, RTA – retrolateral tibial apophysis, rTA – retrolateral tegular apophysis, SD – sperm duct, VTA – ventral tibial apophysis.

**Figure 13.**
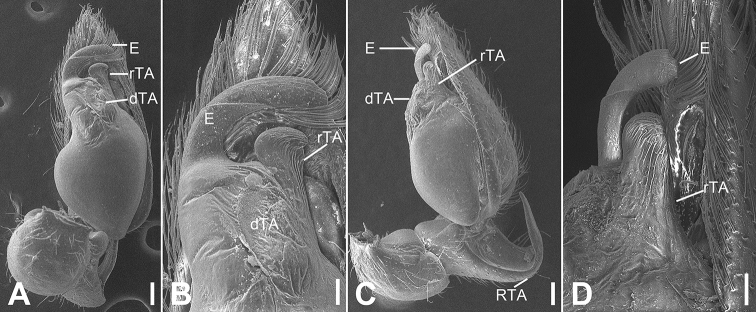
SEM micrographs of *Otacilia wugongshanica* sp. nov., palp of male holotype. **A** ventral view **B** same, detail of embolus, distal tegular apophysis and retrolateral tegular apophysis **C** vento-retrolateral view **D** same, detail of embolus and retrolateral tegular apophysis. Scale bars: 0.1 mm (**A, C**), 40 µm (**B, D**). Abbreviations: dTA – distal tegular apophysis, E – embolus, RTA – retrolateral tibial apophysis, rTA – retrolateral tegular apophysis.

**Figure 14.**
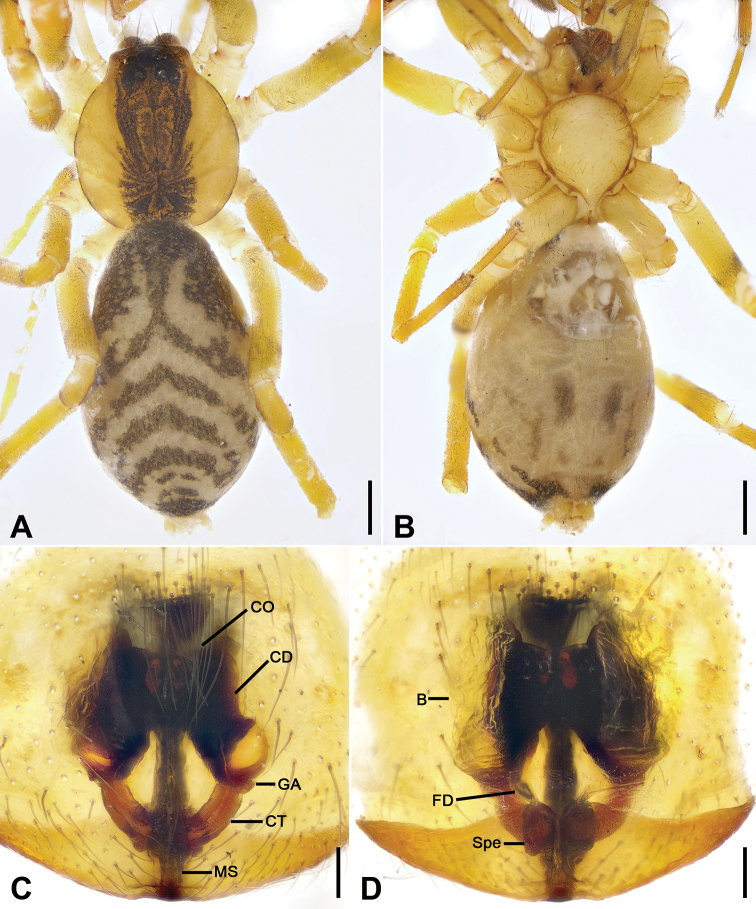
*Otacilia wugongshanica* sp. nov., female paratype. **A** habitus, dorsal view **B** same, ventral view **C** epigyne, ventral view **D** epigyne, dorsal view. Scale bars: 0.5 mm (**A, B**), 0.1 mm (**C, D**). Abbreviations: B – bursa, CD – copulatory duct, CO – copulatory opening, CT – connecting tube, FD – fertilization ducts, GA – glandular appendage, MS – median septum, Spe – spermathecae.

Figures 12 , 13 , 14 , 22

##### Type material.

***Holotype***: ♂, China, Jiangxi Province, Ji’an City, Anfu County, Taishan Town, Wugong Mt., near the ticket office, 27°27'10.79"N, 114°11'8.24"E, 4 January 2020, leg. Ke-ke Liu et al. ***Paratypes***: 2 ♂, 4 ♀, with same data as holotype; 3 ♀, 27°28'25.57"N, 114°12'39.24"E, 633 m, other data as holotype; 3 ♀, 27°28'07.98"N, 114°12'09.55"E, 800 m, other data as holotype; 2 ♂, 1 ♀, Anfu County, Taishan Town, Wenshan Village, Yangshimu Scenic Spot, Grand Canyon, 27°31'43.36"N, 114°14'32.97"E, 552 m, 5 January 2020, leg. Ke-ke Liu et al.

##### Etymology.

The specific name refers to the type locality, Wugongshan; adjective.

##### Diagnosis.

The males of the new species are similar to *Otacilia daweishan* Liu, Xu, Xiao, Yin & Peng, 2019 in having a strong hook-shaped embolus, thick retrolateral tegular apophysis and a finger-like retrolateral tibial apophysis (see Liu et al. 2019: 441, fig. 3B−D), but can be separated from it by the distal tegular apophysis with an oval base (Figs 12D, 13A, B) (vs. with a round base and a mastoid-shaped retrolateral part), the V-shaped sperm duct (Figs 12D, E, 13A, B) (vs. C-shaped) and the retrolateral tibial apophysis with a sharply narrowed basal part (Fig. 12F) (vs. gradually narrowed basal part). The females can be distinguished from *O. daweishan* (see Liu et al. 2019: 441, fig. 4B, C) by the narrowed median septum (Fig. 14C) (vs. broad, sub-triangular median septum) and the transverse epigynal sclerotized ridge (Fig. 14C) (vs. M-shaped).

##### Description.

Male (holotype). Habitus as in Fig. 12A, B. Total length 3.52, carapace 1.74 long, 1.50 wide. Eye sizes and interdistances: AME 0.08, ALE 0.08, PME 0.07, PLE 0.09, AME−AME 0.05, AME−ALE 0.03, PME−PME 0.13, PME−PLE 0.07, AME−PME 0.10, AME−PLE 0.18, ALE−ALE 0.26, PLE−PLE 0.44, ALE−PLE 0.10. MOA 0.25 long, frontal width 0.21, posterior width 0.29. Cervical groove and fovea distinct. Chelicerae (Fig. 12A, B) with three promarginal (middle largest, distal smallest) and six retromarginal teeth (distal largest, third smallest). Sternum (Fig. 12B) with blunt posterior end. Abdomen (Fig. 12A, B) 1.73 long, 1.16 wide, weak dorsal scutum in anterior half. Leg measurements: I 7.31 (1.83, 0.60, 2.27, 1.80, 0.81); II 5.91 (1.55, 0.52, 1.64, 1.40, 0.80); III 5.00 (1.21, 0.50, 1.19, 1.28, 0.82); IV 7.72 (2.07, 0.60, 1.85, 2.25, 0.95). Leg spination (Fig. 12A, B): femur I with two dorsal spines, femora II−IV with one dorsal spine each; femora I pv1111, II pv11; tibiae I v222222222, II v2222222; metatarsi I v2222, II v222.

Colouration (Fig. 12A, B). Carapace yellow, with radial irregular dark stripes medially and arch-shaped dark stripes around margin. Chelicerae yellow-brown. Endites yellow. Labium yellow-brown. Sternum yellow. Legs yellow, with blackish-brown annulations on distal part of femora and tibiae. Abdomen dark brown, with pair of oval and pair of large irregular yellowish spots on posterior of dorsal scutum, three light chevron-shaped stripes in posterior part, and yellowish arch-shaped stripe in front of anal tubercle; venter with sub-trapezoid blackish-brown spot posteromedially and pair of sloping blackish-brown stripes posterolaterally.

Palp (Figs 12C−F, 13). Femoral apophysis well-developed, width less than half of its length. Patella unmodified. Retrolateral tibial apophysis large, longer than tibia, finger-like, bending inwards towards the base of cymbium, with sharply narrowed basal part and slightly blunt tip. Ventral tibial apophysis small, blunt. Sperm duct V-shaped, strongly sclerotized, around base of retrolateral tegular apophysis, distal tegular apophysis and embolus. Retrolateral tegular apophysis clavate, slightly shorter than embolus, apex slightly curved. Distal tegular apophysis triangular, with oval base, covering half of retrolateral tegular apophysis. Embolus thick, hook-shaped, with broad base and blunt tip.

Female (paratype). Habitus as in Fig. 14A, B. Darker than males (Fig. 14A, B). Total length 4.31, carapace 1.55 long, 1.53 wide. Eye sizes and interdistances: AME 0.09, ALE 0.1, PME 0.07, PLE 0.08, AME−AME 0.06, AME−ALE 0.03, PME−PME 0.14, PME−PLE 0.08, AME−PME 0.11, AME−PLE 0.19, ALE−ALE 0.28, PLE−PLE 0.42, ALE−PLE 0.12. MOA 0.25 long, frontal width 0.22, posterior width 0.29. Chelicerae (Fig. 14A, B) with three promarginal (middle largest, distal smallest) and seven retromarginal teeth (distal largest, 6^th^ smallest). Abdomen (Fig. 14A, B) 2.58 long, 1.80 wide. Leg measurements (Fig. 14A, B): I 6.59 (1.70, 0.63, 2.07, 1.60, 0.59); II 5.86 (1.49, 0.62, 1.64, 1.35, 0.76); III 3.91 (1.03, 0.45, 0.87, 0.95, 0.61); IV 7.34 (1.86, 0.59, 1.82, 2.09, 0.98). Leg spination: femora I pv1111, II pv111; tibiae I v22222222, II v2222222.

Colouration (Fig. 14A, B). Abdomen dark brown, with pair of L-shaped yellowish stripes anteriorly and broad arc-shaped mottled stripes posteriorly.

Epigyne (Fig. 14C, D). Epigynal plate bow-shaped, anteriorly with transverse sclerotized ridge and strongly sclerotized fovea, anteromedially with pair of oval copulatory openings, posteromedially with narrowed median septum. Copulatory ducts, glandular appendages and connecting tubes distinctly visible through integument in intact epigyne. Copulatory ducts broad, slightly sloping, located between copulatory openings and glandular appendages, posteriorly with pair of large, bean-shaped transparent bursae. Glandular appendages short, partly covered by bursae, located on anterior of connecting tubes. Connecting tubes shorter than copulatory ducts, posterior part convergent. Spermathecae oval, touching. Fertilization ducts short, located apically on spermathecae, directed anterolaterally.

##### Distribution.

Known only from the type locality in Jiangxi Province, China (Fig. 22).

#### Otacilia yusishanica

Taxon classificationAnimaliaAraneaePhrurolithidae

Liu sp. nov.

A677350A-5529-5307-9C10-8A08DDB4EB01

http://zoobank.org/0CD9F210-2A0D-4292-94F6-76F55F8EA7D2

**Figure 15.**
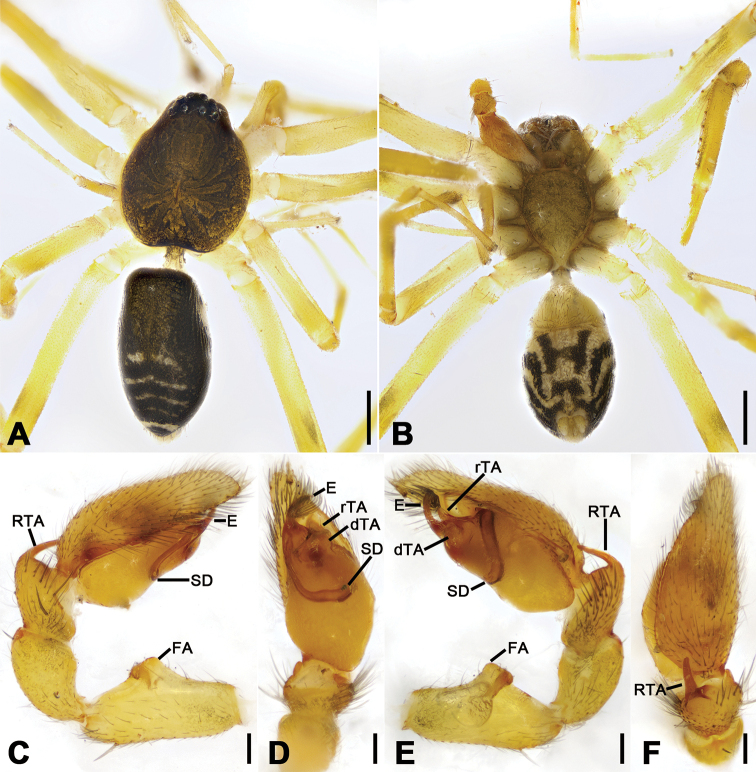
*Otacilia yusishanica* sp. nov., male holotype. **A** habitus, dorsal view **B** same, ventral view **C** palp, prolateral view **D** same, ventral view **E** same, ventro-retrolateral view **F** same, dorsal view. Scale bars: 0.5 mm (**A, B**), 0.1 mm (**C–F**). Abbreviations: dTA – distal tegular apophysis, E – embolus, FA – femoral apophysis, RTA – retrolateral tibial apophysis, rTA – retrolateral tegular apophysis, SD – sperm duct.

**Figure 16.**
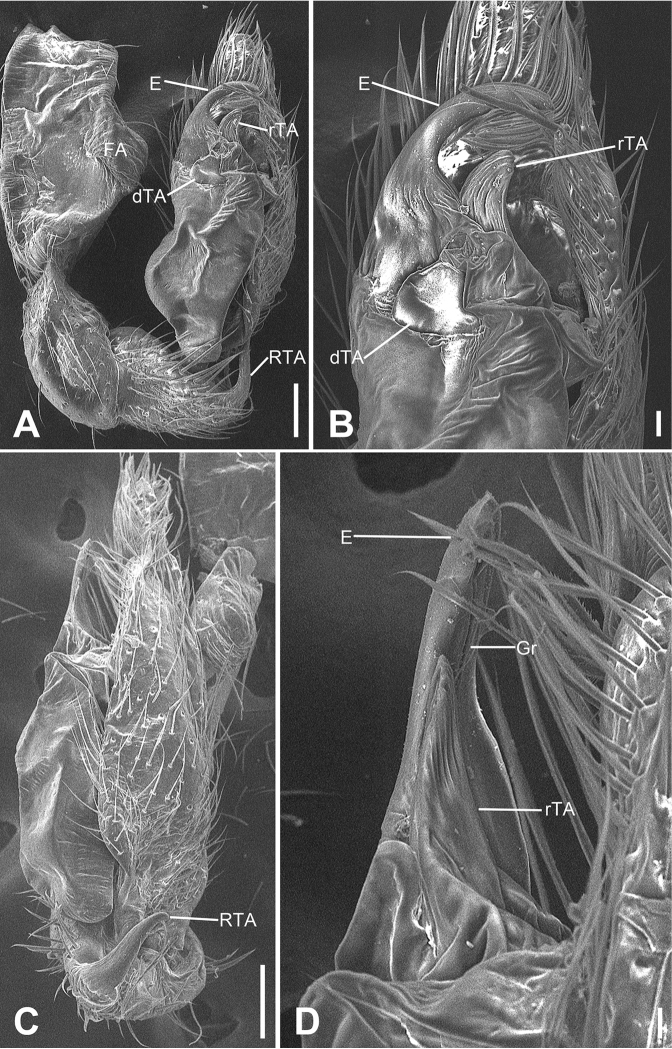
SEM micrographs of *Otacilia yusishanica* sp. nov., palp of male holotype. **A** ventral view **B** same, detail of embolus, distal tegular apophysis and retrolateral tegular apophysis **C** retro-dorsal view **D** same, detail of embolus, embolic groove and retrolateral tegular apophysis. Scale bars: 0.1 mm (**A, C**), 20 µm (**B**), 10 µm (**D**). Abbreviations: dTA – distal tegular apophysis, E – embolus, Gr – groove, RTA – retrolateral tibial apophysis, rTA – retrolateral tegular apophysis.

**Figure 17.**
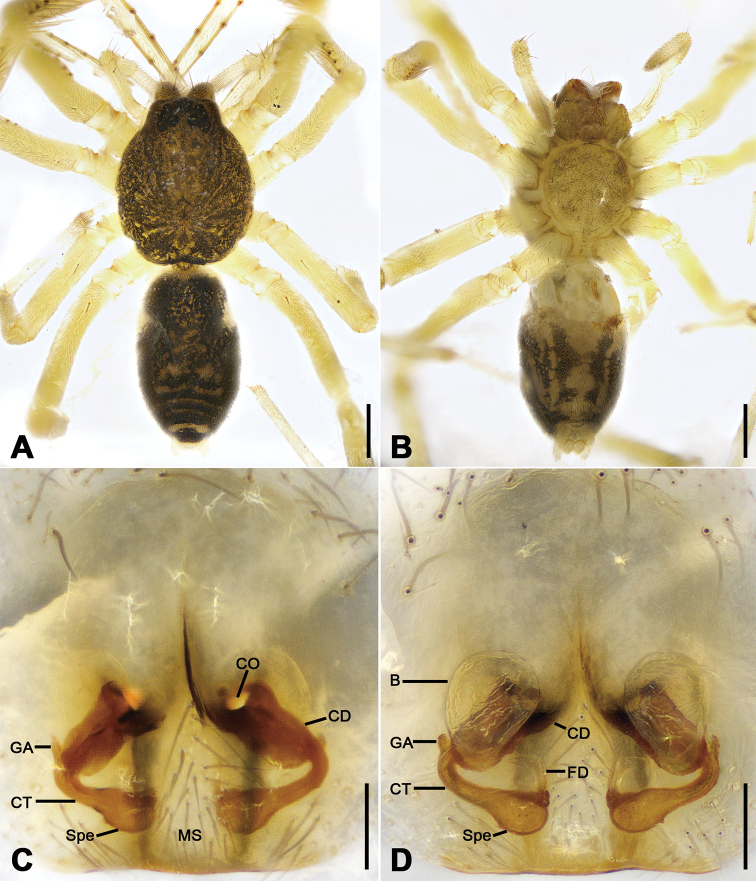
*Otacilia yusishanica* sp. nov., female paratype. **A** habitus, dorsal view **B** same, ventral view **C** epigyne, ventral view **D** epigyne, dorsal view. Scale bars: 0.5 mm (**A, B**), 0.1 mm (**C, D**). Abbreviations: B – bursa, CD – copulatory duct, CO – copulatory opening, CT – connecting tube, FD – fertilization ducts, GA – glandular appendage, MS – median septum, Spe – spermathecae.

Figures 15 , 16 , 17 , 22

##### Type material.

***Holotype***: ♂, China, Jiangxi Province, Ji’an City, Xiajiang County, Yusi Mt., 27°33'05.52"N, 115°16'16.88"E, 202 m, 7 October 2019, leg. Ke-ke Liu et al. ***Paratypes***: 5 ♂, 4 ♀, 4 juveniles, with same data as holotype.

##### Etymology.

The specific name refers to the type locality, Yusishan; adjective.

##### Diagnosis.

The males of the new species are similar to *Otacilia acutangula* Liu, 2020 in having a thick hook-shaped embolus, a C-shaped sperm duct and a finger-like retrolateral tibial apophysis (see Liu et al. 2020: 13, fig. 7C−F), but can be separated from it by the retrolateral tibial apophysis with a straight tip in retrolateral view (Figs 15D, E, 16A, B, D) (vs. with a slightly curved tip). The females resemble *O. acutangula* in having small bursae and thin connecting tubes (see Liu et al. 2020: 13, fig. 8C, D), but can be distinguished from it by the rectangular median septum (Fig. 17C) (vs. triangular) and the widely separated spermathecae (Fig. 17D) (vs. proximate spermatheca).

##### Description.

Male (holotype). Habitus as in Fig. 15A, B. Total length 3.29, carapace 1.48 long, width 1.21 wide. Eye sizes and interdistances: AME 0.07, ALE 0.08, PME 0.06, PLE 0.07, AME−AME 0.05, AME−ALE 0.02, PME−PME 0.11, PME−PLE 0.06, AME−PME 0.09, AME−PLE 0.17, ALE−ALE 0.23, PLE−PLE 0.37, ALE−PLE 0.10. MOA 0.24 long, frontal width 0.20, posterior width 0.25. Chelicerae (Fig. 15A, B) with three promarginal (proximal largest, distal smallest) and six retromarginal teeth (distal larger, others equal in size). Sternum (Fig. 15B) longer than wide. Pedicel 0.28 long. Abdomen (Fig. 15A, B) 1.58 long, 0.92 wide. Leg measurements: I 6.45 (1.65, 0.51, 1.94, 1.53, 0.82); II 5.13 (1.28, 0.53, 1.39, 1.18, 0.75); III 4.35 (1.12, 0.46, 0.94, 1.18, 0.65); IV 7.05 (1.92, 0.57, 1.67, 1.89, 1.00). Leg spination (Fig. 15A, B): femora I−IV with one dorsal spine each; femora I pv1111, II pv11; tibiae I v2222222, II v2222222; metatarsi I v2222, II v222.

Colouration (Fig. 15A, B). Carapace yellow-brown, with irregular, dark yellow, radial strips mediolaterally. Fovea distinct, black. Chelicerae, endites, labium, and sternum yellow-brown. Legs yellow, without dark annulations. Abdomen dark brown, with pair of pale stripes located at posterior of dorsal scutum, three light chevron-shaped stripes in posterior part and one yellowish arc-shaped stripe in front of anal tubercle; venter with H-shaped blackish-brown stripe posteromedially, pair of sloping blackish-brown stripes posterolaterally and N-shaped blackish-brown stripe posteriorly.

Palp (Figs 15C−E, 16). Femoral apophysis well-developed, width more than half of its length. Patella unmodified. Retrolateral tibial apophysis small, less than tibia length, bending inward toward base of cymbium, with straight tip in retrolateral view. Sperm duct C-shaped, strongly sclerotized, around base of subterminal apophysis and embolus. Distal tegular apophysis, membranous, extruding retrolaterally, covering most of retrolateral tegular apophysis. Embolus spine-like, thick, with broad base and blunt apex, embolic groove narrowed.

Female (paratype). Habitus as in Fig. 17A, B. Lighter than males. Total length 3.30, carapace 1.57 long, 1.35 wide. Eye sizes and interdistances: AME 0.07, ALE 0.06, PME 0.05, PLE 0.07, AME−AME 0.07, AME−ALE 0.04, PME−PME 0.10, AME−PME 0.10, AME−PLE 0.18, ALE−ALE 0.24, PLE−PLE 0.36, ALE−PLE 0.12. MOA 0.23 long, frontal width 0.19, posterior width 0.25. Chelicerae (Fig. 17A, B) with three promarginal (proximal largest, distal smallest) and five retromarginal teeth (distal largest, second smallest, all teeth with a same base). Pedicel 0.10 long. Abdomen (Fig. 17A, B) 1.63 long, 1.00 wide. Leg measurements (Fig. 17A, B): I 6.87 (1.87, 0.61, 2.05, 1.61, 0.73); II 5.00 (1.27, 0.47, 1.41, 1.15, 0.70); III 4.49 (1.16, 0.51, 0.99, 1.06, 0.77); IV 7.09 (1.82, 0.61, 1.73, 2.00, 0.93). Leg spination (Fig. 17A, B): femora I pv1111, II pv111; tibiae I v22222222, II v22222222; metatarsi I v2222, II v2222.

Colouration (Fig. 17A, B). Darker than males. Abdomen, venter with two pairs of dark brown stripes posteriorly, median one touching.

Epigyne (Fig. 17C, D). Epigynal plate bow-like, anterior margin weakly sclerotized, arc-shaped, medially with pair of hole-shaped copulatory openings, posteriorly with rectangular median septum. Copulatory ducts, glandular appendages, connecting tubes and spermathecae distinctly visible through integument in intact epigyne. Copulatory ducts between copulatory openings and glandular appendages, sloping laterally, broad, short, posteriorly with pair of small, oval, transparent bursae. Glandular appendages short, located on anterior of connecting tubes, near base of bursae. Connecting tubes slightly shorter than copulatory ducts, slightly curved forwards. Spermathecae slightly expanded, elongated, separated by mark of median septum. Fertilization duct short, directed anterolaterally.

##### Distribution.

Known only from the type locality in Jiangxi Province, China (Fig. 22).

#### Otacilia zaoshiica

Taxon classificationAnimaliaAraneaePhrurolithidae

Liu sp. nov.

88AD3E16-A895-53FB-A488-C2AA7F33F064

http://zoobank.org/DA024E13-9A9F-412F-AFCB-93C9906AA61F

**Figure 18.**
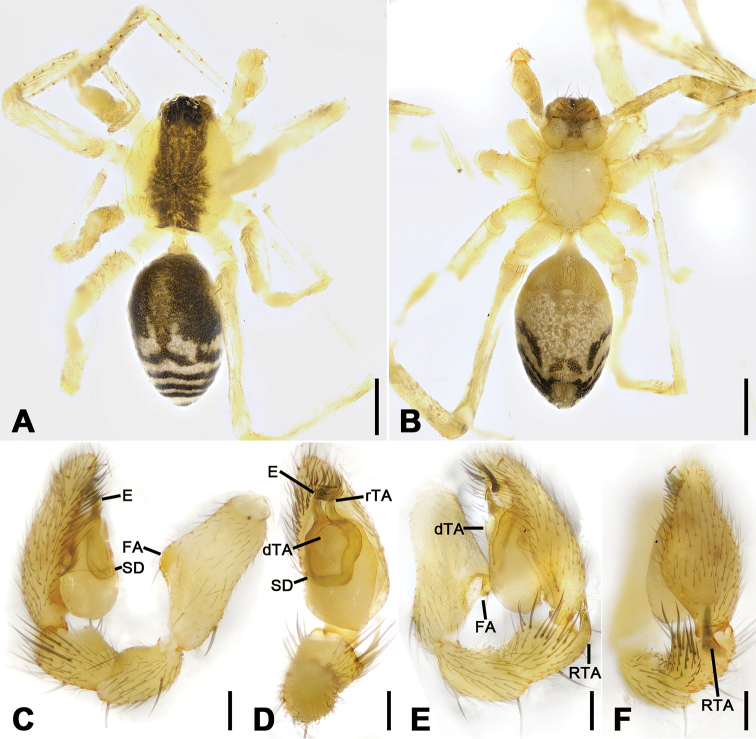
*Otacilia zaoshiica* sp. nov., male holotype. **A** habitus, dorsal view **B** same, ventral view **C** palp, prolateral view **D** same, ventral view **E** same, ventro-retrolateral view **F** same, dorsal view. Scale bars: 0.5 mm (**A, B**), 0.1 mm (**C–F**). Abbreviations: dTA – distal tegular apophysis, E – embolus, FA – femoral apophysis, RTA – retrolateral tibial apophysis, rTA – retrolateral tegular apophysis, SD – sperm duct.

**Figure 19.**
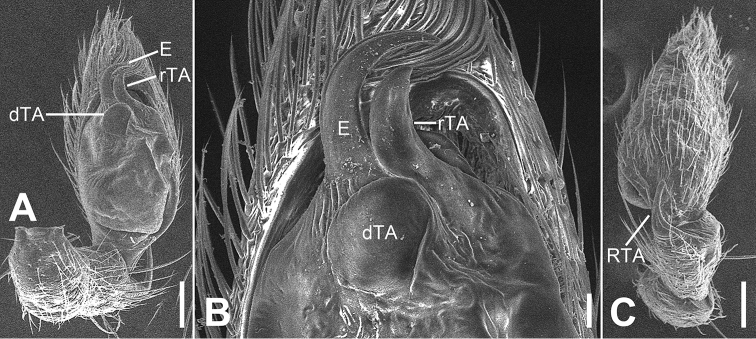
SEM micrographs of *Otacilia zaoshiica* sp. nov., palp of male holotype. **A** ventral view **B** same, detail of embolus, distal tegular apophysis and retrolateral tegular apophysis **C** dorsal view. Scale bars: 0.1 mm (**A, C**), 20 µm (**B**). Abbreviations: dTA – distal tegular apophysis, E – embolus, RTA – retrolateral tibial apophysis, rTA – retrolateral tegular apophysis.

**Figure 20.**
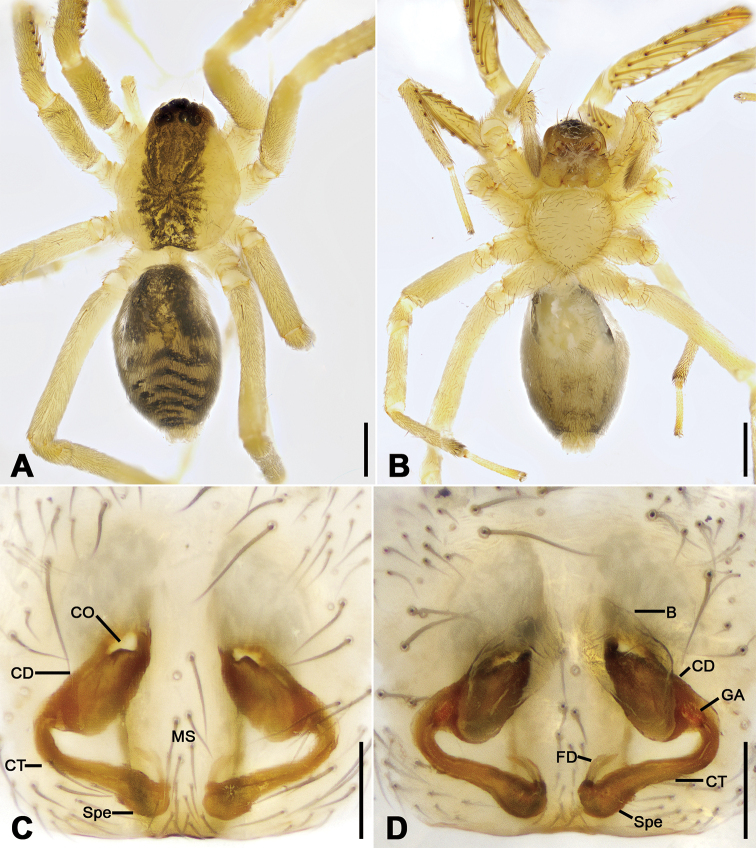
*Otacilia zaoshiica* sp. nov., female paratype. **A** habitus, dorsal view **B** same, ventral view **C** epigyne, ventral view **D** epigyne, dorsal view. Scale bars: 0.5 mm (**A, B**), 0.1 mm (**C, D**). Abbreviations: B – bursa, CD – copulatory duct, CO – copulatory opening, CT – connecting tube, FD – fertilization ducts, GA – glandular appendage, MS – median septum, Spe – spermathecae.

Figures 18 , 19 , 20 , 22

##### Type material.

***Holotype***: ♂, China, Jiangxi Province, Ji’an City, Xingan County, Zaoshi Village, 27°46'15.63"N, 115°39'38.10"E, 589 m, 7 October 2019, leg. Ke-ke Liu et al. ***Paratypes***: 4 ♂, 1 ♀, 4 juveniles, with same data as holotype.

##### Etymology.

The specific name is derived from the type locality, Zaoshi village, which is one of the famous traditional villages; adjective.

##### Diagnosis.

The males of the new species are similar to *O. yusishanica* sp. nov. described above in having a finger-like retrolateral tibial apophysis (Fig. 15C−F), but can be separated from it by the arc-shaped embolic base (Figs 18D, 19A, B) (vs. triangular) and the retrolateral tegular apophysis accompanied by the embolus (Figs 18D, 19A, B) (vs. separated).The females resemble *O. yusishanica* sp. nov. by having a broad median septum and the thin connecting tubes (Fig. 17C, D), but can be separated from it by the saddle-shaped copulatory openings (Fig. 20C) (vs. oval) and the spermathecae separated by approximately 1/3 of the median septum width (Fig. 20D) (vs. 2/3).

##### Description.

Male (holotype). Habitus as in Fig. 18A, B. Total length 2.90, carapace 1.21 long, width 1.03 wide. Eye sizes and interdistances: AME 0.05, ALE 0.06, PME 0.05, PLE 0.06, AME−AME 0.07, AME−ALE 0.03, PME−PME 0.12, PME−PLE 0.05, AME−PME 0.10, AME−PLE 0.12, ALE−ALE 0.21, PLE−PLE 0.31, ALE−PLE 0.1. MOA 0.20 long, frontal width 0.15, posterior width 0.19. Chelicerae (Fig. 18A, B) with three promarginal (proximal largest, second smallest) and five retromarginal teeth (distal largest, second smallest). Sternum (Fig. 18B) longer than wide. Pedicel 0.21 long. Abdomen (Fig. 18A, B) 1.51 long, 0.86 wide. Leg measurements: I 4.92 (1.24, 0.41, 1.50, 1.06, 0.71); II 3.86 (1.08, 0.45, 1.04, 0.99, 0.30); III 3.11 (0.84, 0.31, 0.69, 0.83, 0.44); IV 5.75 (1.64, 0.43, 1.37, 1.59, 0.72). Leg spination (Fig. 18A, B): femora I−IV with one dorsal spine each; femora I pv1111, II pv11; tibiae I v2222222, II v2222222; metatarsi I v2222, II v2222.

Colouration (Fig. 18A, B). Carapace yellow, medially with broad dark brown mottled markings. Fovea distinct, black. Chelicerae, endites, labium and sternum yellow-brown. Legs yellow, without dark annulations. Abdomen dark brown, with pair of round and oval pale spots located at posterior of dorsal scutum and three light chevron-shaped stripes in posterior part, and one yellowish transversal stripe in front of anal tubercle.

Palp (Figs 18C−F, 19). Femoral apophysis well-developed, width more than half of its length. Patella unmodified. Retrolateral tibial apophysis large, longer than tibia, sword-like in ventral view, bending inward to base of cymbium, medial part widened and slightly curved, with strong spine-like tip. Sperm duct U-shaped, strongly sclerotized, around base of subterminal apophysis, distal tegular apophysis and embolus. Retrolateral tegular apophysis straight, broad, as long as embolus, anteriorly widened. Distal tegular apophysis membranous, fan-shaped, extending to median bulb. Embolus thick, hook-shaped, with broad base and blunt tip. Embolus relatively long, thick spine-like, with broad base and blunt apex.

Female (paratype). Habitus as in Fig. 20A, B. Lighter than males. Total length 3.07, carapace 1.36 long, 1.19 wide. Eye sizes and interdistances: AME 0.09, ALE 0.09, PME 0.08, PLE 0.08, AME−AME 0.04, AME−ALE 0.01, PME−PME 0.09, PME−PLE 0.06, AME−PME 0.06, AME−PLE 0.14, ALE−ALE 0.21, PLE−PLE 0.32, ALE−PLE 0.09. MOA 0.24 long, frontal width 0.20, posterior width 0.24. Chelicerae (Fig. 20A, B) with three promarginal (proximal largest, distal smallest) and six retromarginal teeth (distal largest, second smallest). Pedicel 0.17 long. Abdomen (Fig. 19A, B) 1.47 long, 0.97 wide. Leg measurements (Fig. 20A): I 5.63 (1.47, 0.51, 1.73, 1.25, 0.67); II 4.50 (1.20, 0.46, 1.25, 1.02, 0.57); III 3.84 (1.02, 0.43, 0.87, 0.93, 0.59); IV 6.14 (1.69, 0.55, 1.44, 1.71, 0.75). Leg spination (Fig. 20A, B): femora I pv1111, II pv111; tibiae I v22222222, II v2222222; metatarsi I v2222, II v222.

Epigyne (Fig. 20C, D). Epigynal plate mask-shaped, anterior margin slightly sclerotized, transverse, medially with pair of touching saddle-shaped copulatory openings, posteriorly with sub-trapezoidal median septum. Copulatory ducts, connecting tubes and spermathecae distinctly visible through integument in intact epigyne. Copulatory ducts between copulatory openings and glandular appendages, sloping laterally, proper broad, posteriorly with pair of large, oval, transparent bursae. Glandular appendages short, near the base of bursae. Connecting tubes slightly shorter than copulatory ducts, slightly curved backwards. Spermathecae slightly expanded, directed medially, separated by approximately 1/3 of median septum width. Fertilization ducts short, with semi-ovoid base, directed forward.

##### Distribution.

Known only from the type locality in Jiangxi Province, China (Fig. 22).

#### Otacilia ziyaoshanica

Taxon classificationAnimaliaAraneaePhrurolithidae

Liu sp. nov.

91386AB9-6530-5FF7-B26B-53FF7AB89BEE

http://zoobank.org/A2E72C0B-7B5C-4251-A47D-44451406E9C7

Figures 21 , 22

##### Type material.

***Holotype***: ♀, China, Jiangxi Province, Ji’an City, Taihe County, Ziyao Mt., 26°42'49.38"N, 115°13'32.82"E, 198 m, 6 October 2019, leg. Ke-ke Liu et al.

##### Etymology.

The specific name refers to the type locality, Ziyaoshan; adjective.

##### Diagnosis.

**Figure 21.**
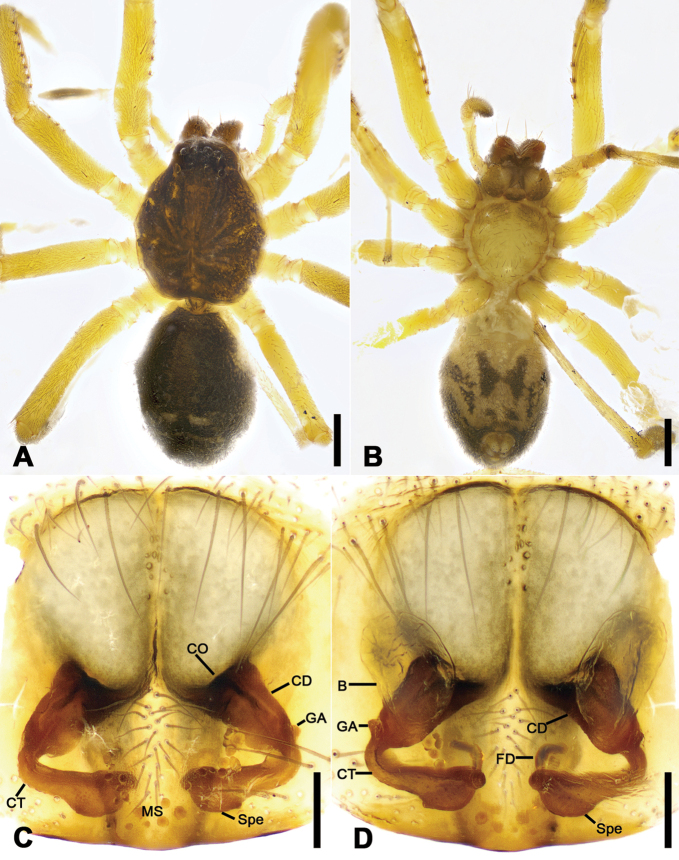
*Otacilia ziyaoshanica* sp. nov., female paratype. **A** habitus, dorsal view **B** same, ventral view **C** epigyne, ventral view **D** epigyne, dorsal view. Scale bars: 0.5 mm (**A, B**), 0.1 mm (**C, D**). Abbreviations: B – bursa, CD – copulatory duct, CO – copulatory opening, CT – connecting tube, FD – fertilization ducts, GA – glandular appendage, MS – median septum, Spe – spermathecae.

The female of this species is similar to *Otacilia acutangula* Liu, 2020 and *O. macrospora* Fu, Zhang & Zhang, 2016 in having a M-shaped epigynal margin and concave anterior epigynal part (see Liu et al. 2020: 13, fig. 8C, D; Fu et al. 2016: 138, fig. 20, 21), but can be separated from them by the chelicerae with three retromarginal teeth (Fig. 21B) (vs. five in *O. acutangula* and *O. macrospora*) and the widely separated spermathecae (Fig. 21D) (vs. slightly separated in *O. acutangula* and *O. macrospora*).

##### Description.

Female. Habitus as in Fig. 21A, B. Total length 3.45, carapace 1.60 long, 1.31 wide. Eye sizes and interdistances: AME 0.1, ALE 0.08, PME 0.06, PLE 0.08, AME−AME 0.05, AME−ALE 0.02, PME−PME 0.12, PME−PLE 0.06, AME−PME 0.08, AME−PLE 0.16, ALE−ALE 0.25, PLE−PLE 0.37, ALE−PLE 0.1. MOA 0.23 long, frontal width 0.23, posterior width 0.25. Chelicerae (Fig. 21A, B) with three promarginal (proximal largest, distal smallest) and three retromarginal teeth (distal largest, third smallest). Sternum (Fig. 21B), posteriorly proper blunt. Pedicel 0.14 long. Abdomen (Fig. 18A, B) 1.73 long, 1.20 wide. Leg measurements: I 6.20 (1.41, 0.57, 1.96, 1.50, 0.76); II 4.98 (1.07, 0.55, 1.51, 1.10, 0.75); III 4.39 (1.17, 0.39, 0.97, 1.17, 0.69); IV 6.63 (1.76, 0.59, 1.68, 1.64, 0.96). Leg spination (Fig. 18A, B): femora I−IV with 1 dorsal spine each; femora I p11111, p1111 (right), II p11; tibiae I v22222222, II v22222222; metatarsi I v2222, II v2222.

Colouration (Fig. 21A, B). Carapace yellow, with radial, irregular dark stripes mediolaterally. Sternum yellow, with yellow-brown margin. Legs yellow, without annulations on tibiae and distal part of femora, patellae and metatarsi. Abdomen brown, with abundant yellowish spots in dorsal view.

Epigyne (Fig. 21C, D). Epigynal plate mushroom like, anteriorly with M-shaped sclerotized margin, medially with pair of slit-like copulatory openings covered by epigynal plug, posteromedially with trapezoidal median septum. Copulatory ducts, glandular appendages, connecting tubes and spermathecae distinctly visible through integument in intact epigyne. Copulatory ducts short and broad, posteriorly with pair of large, oval, transparent bursae. Glandular appendages short, located on anterior of connecting tubes, near base of bursae. Connecting tubes longer than copulatory ducts, convergent. Spermathecae slightly expanded, separated by approximately 1/2 of median septum width. Fertilization ducts short, directed antero-laterally.

Male unknown.

##### Distribution.

**Figure 22.**
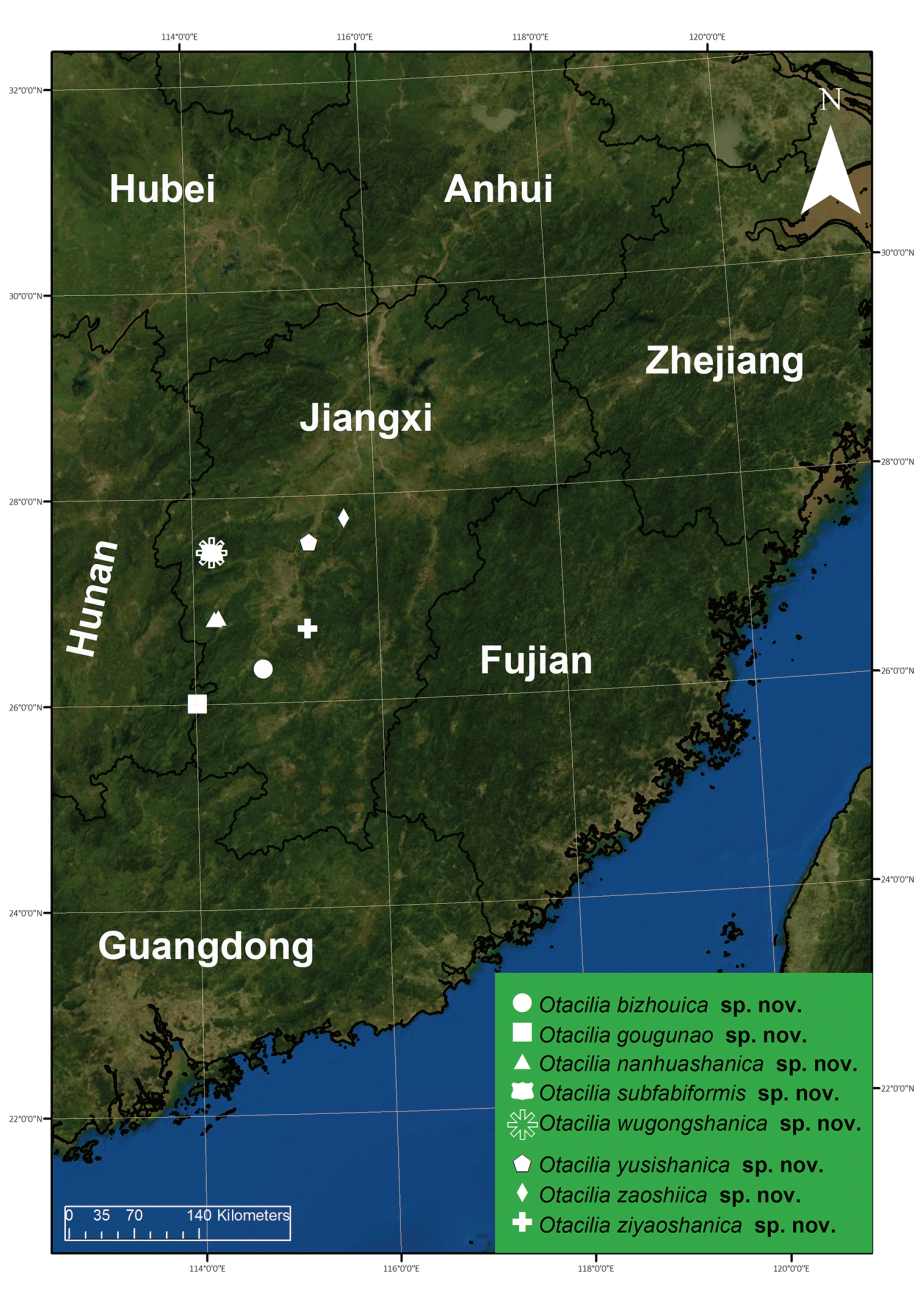
Records of *Otacilia bizhouica* sp. nov., *O. gougunao* sp. nov., *O. nanhuashanica* sp. nov., *O. subfabiformis* sp. nov., *O. wugongshanica* sp. nov., *O. yusishanica* sp. nov., *O. zaoshiica* sp. nov. and *O. ziyaoshanica*

Known only from the type locality in Jiangxi Province, China (Fig. 22).

## Discussion

At present, the genus *Otacilia* has an exclusively Asian distribution, with the highest number of species found in the subtropical and tropical areas of China. Up to now, 89 species of *Otacilia* are known from China, including the eight new species described above (WSC 2020). During the past six years, we focused on the sac spiders of South China, in areas such as the Guangxi Zhuang Autonomous Region, Guang Dong Province, Hunan Province and Jiangxi Province, and found that most *Otacilia* species live in mountainous regions and hills over 200 metres above sea level (Liu et al. 2019, 2020). Ji’an City is located at the middle section of the Luoxiao Mountains in South China, which is mainly surrounded by hilly topography. Many new *Otacilia* species have been found and recorded from different mountains in this area (Liu et al. 2019, 2020). It is interesting to note that these new species, including those newly described here, clearly appear on different mountains or different aspects of a single mountain. It is likely that the total number of known species of *Otacilia* will rapidly rise to 100 by the end of 2021, as our survey is focused on sac spiders. These results suggest that Jiangxi Province has a spectacular diversity of *Otacilia* and that it is necessary to continue surveying it in the future.

## Supplementary Material

XML Treatment for Otacilia bizhouica

XML Treatment for Otacilia gougunao

XML Treatment for Otacilia nanhuashanica

XML Treatment for Otacilia subfabiformis

XML Treatment for Otacilia wugongshanica

XML Treatment for Otacilia yusishanica

XML Treatment for Otacilia zaoshiica

XML Treatment for Otacilia ziyaoshanica
